# IgG4-Related Disease Presenting With Diffuse Coronary Atherosclerosis Characterized by Marked Lipid-Rich Necrotic Core Burden

**DOI:** 10.1016/j.jaccas.2026.107264

**Published:** 2026-03-05

**Authors:** Francisco X. Elisarraras, Robert A. Pelberg, Jacob S. Roberts, Jay A. Shah, Jeffrey Xia, Sammy Sayed, Norman E. Lepor, Jairo Aldana-Bitar, Ronald P. Karlsberg, Geoffrey Cho

**Affiliations:** a University of California–Los Angeles Health, Los Angeles, California, USA; b Elucid Bioimaging, Boston, Massachusetts, USA; c Division of Cardiology, Johns Hopkins Hospital, Baltimore, Maryland, USA; d National Heart Institute, Beverly Hills, California, USA; e Cedars-Sinai Heart Institute, Los Angeles, California, USA; f Cardiovascular Research Foundation of Southern California, Los Angeles, California, USA; g David Geffen School of Medicine at the University of California–Los Angeles, Los Angeles, California, USA

**Keywords:** atherosclerosis, cardiac risk, coronary artery disease (CAD), coronary CT angiography (CCTA), coronary periarteritis, high-risk plaque, IgG4-related disease (IgG4-RD), lipid-rich necrotic core (LRNC), plaque characterization, primary prevention, risk factor, secondary prevention

## Abstract

**Background:**

IgG4-related disease (IgG4-RD) is a systemic fibroinflammatory disorder with multiorgan involvement, including increasingly recognized cardiovascular manifestations such as aortitis and periarteritis. Coronary involvement remains rare and is often clinically silent and poorly characterized, with most reported cases associated with pericoronary inflammation, ectasia, or aneurysmal dilation rather than diffuse high-risk atherosclerosis.

**Case Summary:**

A 63-year-old man with longstanding IgG4-RD presented with abdominal pain and no cardiac symptoms. Computed tomography demonstrated periaortic inflammation, incidental coronary calcification, and markedly elevated serum IgG4. Coronary computed tomography angiography revealed diffuse pericoronary thickening without aneurysmal dilation. Advanced plaque analysis showed an extreme coronary plaque burden dominated by lipid-rich necrotic core and noncalcified plaque, consistent with high-risk atherosclerosis.

**Discussion:**

This case expands the spectrum of IgG4-related coronary involvement, demonstrating diffuse, severe, inflammation-associated atherosclerosis without ectasia or aneurysm and highlighting the disconnect between symptoms and disease burden, with potential for silent, high-risk coronary disease.

**Take-Home Message:**

IgG4-RD may harbor clinically silent but high-risk coronary disease, supporting vigilance, proactive coronary imaging, aggressive immunosuppression, and intensive secondary prevention.

## Visual Summary

**Figure:**
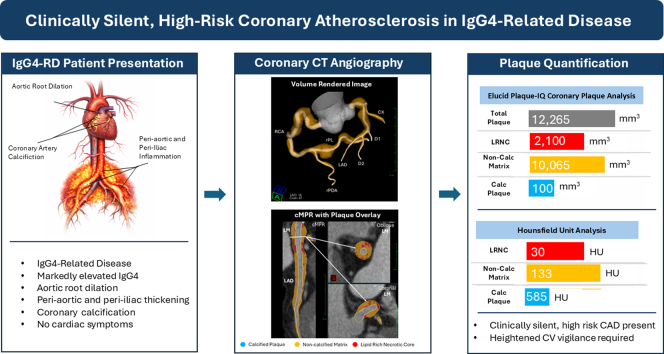

## History of Presentation

A 63-year-old man with established IgG4-related disease (IgG4-RD)^1^ presented with progressive abdominal pain concerning for disease reactivation. He denied chest pain, dyspnea, exertional symptoms, palpitations, syncope, or other cardiovascular issues. There was no prior clinical suspicion for cardiovascular involvement based on symptoms or prior testing.

## Past Medical History

The patient's medical history was notable for biopsy-proven IgG4-RD. Prior treatment included prolonged courses of prednisone, hydroxychloroquine, and 4 cycles of rituximab, with only partial and inconsistent clinical response. He had no known history of cardiovascular disease, coronary artery disease, hypertension, diabetes mellitus, or prior abnormal cardiac imaging.

## Differential Diagnosis

The differential diagnosis for the vascular and coronary findings included IgG4-related periarteritis with coronary involvement, diffuse inflammatory or immune-mediated coronary arteritis, accelerated atherosclerosis related to chronic systemic inflammation, and less likely vasculitides such as Kawasaki disease, Takayasu arteritis, or polyarteritis nodosa. Given the patient's known IgG4-RD with active disease, IgG4-related coronary involvement was strongly suspected.

## Investigations

Computed tomography of the abdomen and pelvis demonstrated extensive soft tissue thickening encasing the abdominal aorta and bilateral iliac arteries, consistent with active periarteritis, as well as incidental coronary artery calcifications and mild ascending aortic ectasia. Serum IgG4 was markedly elevated at 2,436 mg/dL (reference: <123 mg/dL), supporting active systemic disease.

Because IgG4-RD coronary arteritis, while rare, is a recognized manifestation of IgG4-RD,^1^, ^2^, ^3^ and given the presence of coronary artery calcification, coronary computed tomography angiography (CCTA) was pursued to evaluate for occult coronary involvement. CCTA revealed diffuse, circumferential pericoronary arterial thickening involving all major epicardial coronary vessels, without focal stenosis, aneurysm, or ectasia. Comprehensive quantitative and qualitative plaque analysis was performed using PlaqueIQ (Elucid)—a Food & Drug Administration–cleared, histopathologically validated plaque characterization platform.^4^, ^5^, ^6^ This analysis demonstrated an extreme total coronary plaque volume of 12,265 mm^3^, composed predominantly of lipid-rich necrotic core (LRNC) (2,100 mm^3^) and noncalcified matrix (10,065 mm^3^), with minimal calcified plaque (100 mm^3^), consistent with highly active, high-risk coronary atherosclerosis (Table 1).

**Table 1:** Elucid PlaqueIQ Quantification Report According to Plaque Subtype

Territory	Calcified Plaque (mm^3^)	Noncalcified Matrix (mm^3^)	Lipid-Rich Necrotic Core (mm^3^)	Total Plaque Volume (mm^3^)
LM	9.1	696.4	130.1	835.6
LAD	12.6	1,670.2	356.8	2,039.6
LCX	7.2	500.6	97.4	605.3
RCA	71.1	7,198.1	1,515.3	8,784.5
Total	100.0	10,065.3	2,099.6	12,265.0

LAD = left anterior descending artery; LCX = left circumflex artery; LM = left main artery; RCA = right coronary artery.

**Figure 1:**
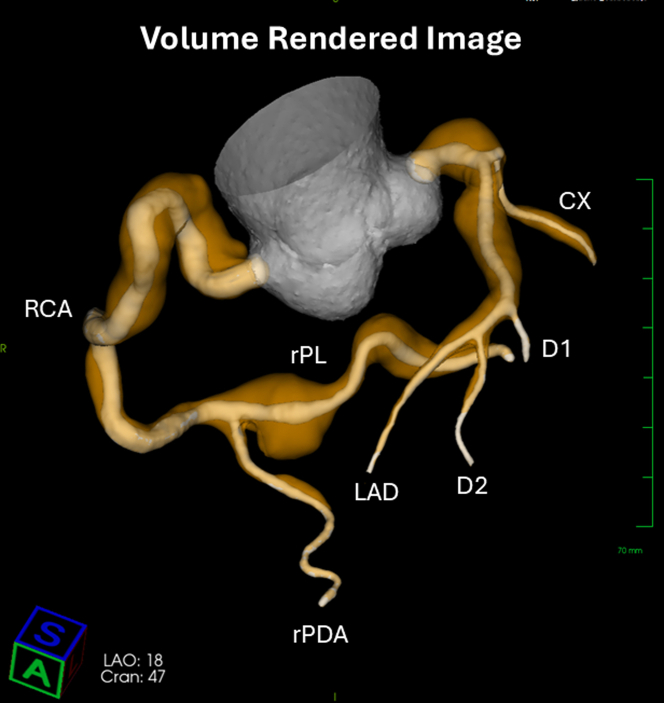
Volume-Rendered CCTA Image Reconstruction Demonstrating Diffuse, Triple-Vessel Coronary Artery Disease CCTA = coronary computed tomography angiography; CX = circumflex artery; D1 = first diagonal branch; D2 = second diagonal branch; LAD = left anterior descending artery; RCA = right coronary artery; rPDA = right posterior descending artery; rPL = right posterolateral branch.

**Figure 2:**
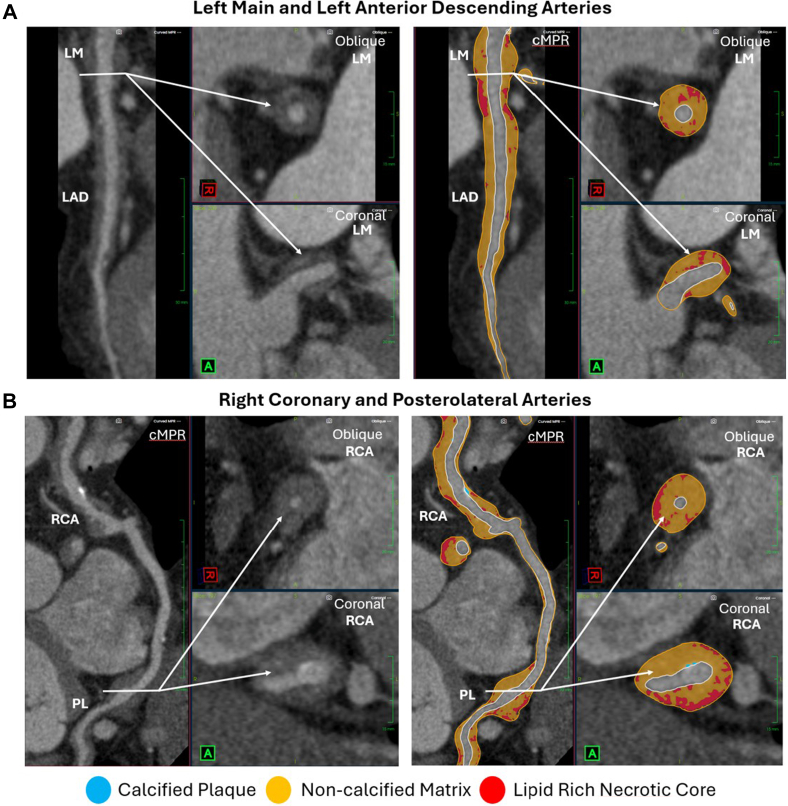
Coronary Plaque Visualization on CCTA With Plaque Subtype Overlay Curved multiplanar reformatted, oblique, and coronal maximum-intensity projections—with and without plaque overlay—of the (A) left main/left anterior descending artery and (B) right coronary artery system demonstrating diffuse coronary plaque with a high volume of lipid-rich necrotic core (red). cMPR = curved multiplanar reformation; LAD = left anterior descending artery; LM = left main artery; PL = posterolateral branch; RCA = right coronary artery.

A volume-rendered reconstruction (Figure 1) demonstrated diffuse triple-vessel coronary artery disease. Multiplanar reformatted views demonstrated extensive, diffuse noncalcified matrix and LRNC—the plaque subtype most strongly associated with plaque vulnerability and acute coronary events^7^—despite the absence of ischemic symptoms. Figure 2 depicts curved multiplanar reformatted, oblique, and coronal depictions—with and without plaque overlay—of the left main/left anterior descending arteries and the right coronary artery/right posterolateral vessel, respectively. No significant coronary ectasia or aneurysmal dilation was identified.

**Figure 3:**
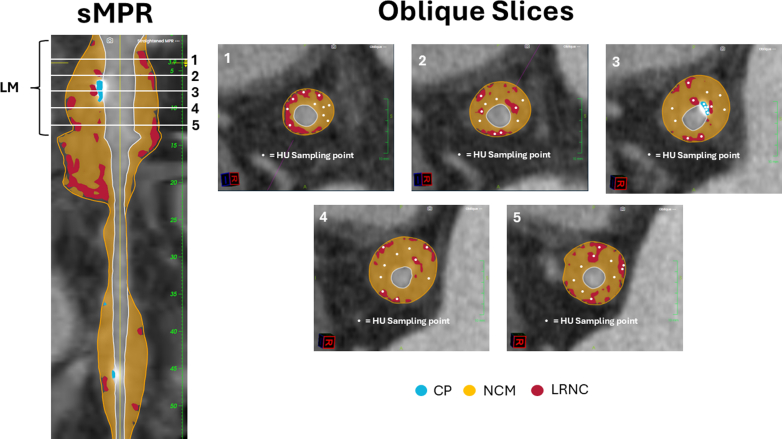
Depiction of the Hounsfield Unit Sampling Methodology CP = calcified plaque; HU = Hounsfield unit; LM = left main artery; LRNC = lipid-rich necrotic core; NCM = noncalcified matrix; sMPR = stretched multiplanar reformation.

**Figure 4:**
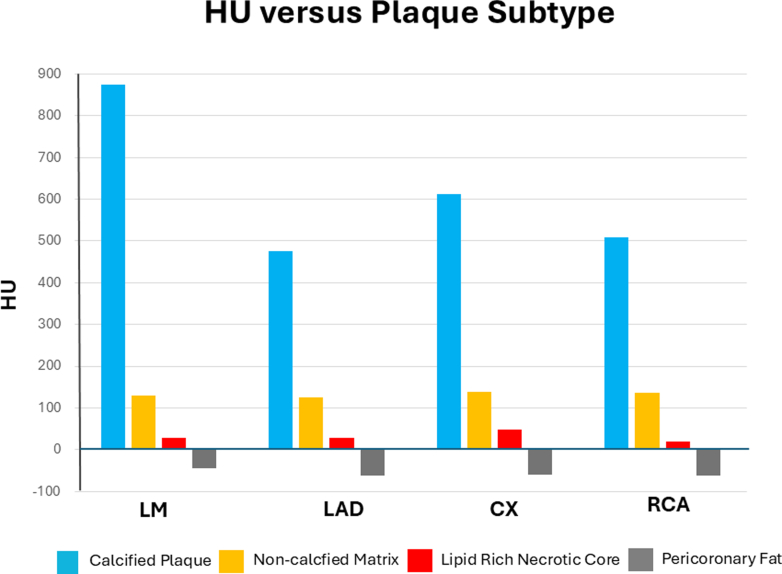
Average Hounsfield Unit Values for Calcified Plaque, Noncalcified Matrix, Lipid-Rich Necrotic Core, and Pericoronary Fat According to Territory CX = circumflex artery; HU = Hounsfield unit; LAD = left anterior descending artery; LM = left main artery; RCA = right coronary artery.

To distinguish plaque from inflamed pericoronary fat, Hounsfield unit (HU) sampling was performed (Figures 3 and 4). In the proximal, mid, and distal segments of the left main artery, left anterior descending artery, right coronary artery, and circumflex artery, 25 HU measurements per segment—5 samples from each of 5 slices—were obtained for calcified plaque (when present), noncalcified matrix, LRNC, and pericoronary fat. Mean attenuation values across coronary segments aligned with established ranges for plaque subtypes and were distinct from pericoronary inflammatory tissue, confirming true coronary atherosclerosis rather than isolated periarterial inflammation. Figure 3 illustrates the sampling methodology, and Figure 4 displays the mean HU values.

## Management

After identification of diffuse high-risk coronary atherosclerosis, high-intensity immunosuppressive therapy was restarted, with close rheumatologic and cardiovascular co-management. Intensive secondary prevention strategies were initiated, including high-intensity statin therapy and optimization of modifiable cardiovascular risk factors. Given the absence of focal obstructive disease or ischemic symptoms, revascularization was not pursued.

## Outcome and Follow-Up

At the short-term follow-up, the patient remained free of cardiovascular symptoms, with improvement in systemic inflammatory markers and stabilization of IgG4 levels. Ongoing surveillance with serial clinical assessment and repeat multimodality cardiovascular imaging was planned to monitor disease activity and coronary plaque burden.

## Discussion

IgG4-RD is increasingly recognized to involve the cardiovascular system; however, coronary manifestations remain uncommon and are typically described in association with pericoronary inflammation, ectasia, or aneurysmal dilation.^2^ This case expands the recognized spectrum of IgG4-related coronary disease by demonstrating diffuse, severe, inflammation-associated coronary atherosclerosis without aneurysm formation, characterized by an exceptionally high burden of LRNC. The absence of ischemic symptoms despite extensive high-risk plaque highlights the potential disconnect between clinical presentation and disease severity and risk in IgG4-RD. To our knowledge, this represents the first documented case of diffuse, severe coronary atherosclerosis in IgG4-RD without aneurysmal dilation, characterized by a markedly elevated burden of LRNC. This case underscores the potential for clinically silent yet high-risk coronary artery disease in IgG4-RD and highlights the need for heightened cardiovascular vigilance, advanced coronary imaging, and precision-guided management.

## Conclusions

This case demonstrates that IgG4-RD can be associated with diffuse, severe, high-risk coronary atherosclerosis in the absence of aneurysmal dilation or ischemic symptoms. Advanced CCTA with quantitative plaque characterization enabled accurate differentiation between pericoronary inflammation and true atherosclerotic disease, revealing an unexpectedly large burden of LRNC. These findings highlight the importance of considering occult coronary involvement in patients with active IgG4-RD and support the role of advanced imaging in guiding risk stratification and management.

**Visual Summary:**
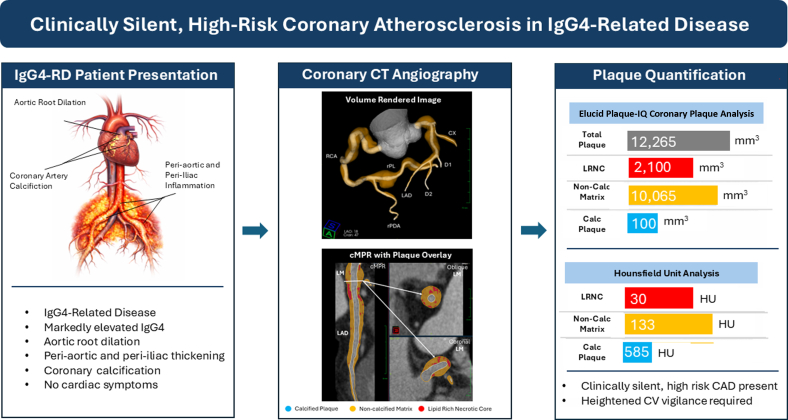
Summary of Clinical Case Progression CAD = coronary artery disease; calc = calcified; cMPR = curved multiplanar reformation; CT = computed tomography; CV = cardiovascular; CX = circumflex artery; D1 = first diagonal branch; D2 = second diagonal branch; HU = Hounsfield unit; IgG4-RD = IgG4-related disease; LAD = left anterior descending artery; LM = left main artery; LRNC = lipid-rich necrotic core; RCA = right coronary artery; rPDA = right posterior descending artery; rPL = right posterolateral branch.

## Funding Support and Author Disclosures

Drs Pelberg and Aldana-Bitar are full-time employees of Elucid Bioimaging and hold of company stock options. All other authors have reported that they have no relationships relevant to the contents of this paper to disclose.

**Take-Home Messages**

• Patients with IgG4-related disease may harbor clinically silent but high-risk coronary artery disease driven by chronic inflammation.
• Proactive cardiovascular assessment using advanced coronary imaging, coupled with aggressive immunosuppression and secondary prevention, should be considered to mitigate the risk of adverse coronary events in this under-recognized population.

**Equipment List:** CCTA Equipment, Protocols, and Plaque Analysis Tools Used

CCTA • Somatom (Siemens) • 64 detector rows • 0.75-mm slice reconstruction at 0.4-mm intervals • Pitch of 0.2 • 130 mL Isovue 370 • kVP • mA
Advanced plaque analysis • PlaqueIQ (Elucid)

CCTA = coronary computed tomography angiography.
